# Comparison of structural variants in the whole genome sequences of two *Medicago truncatula* ecotypes: Jemalong A17 and R108

**DOI:** 10.1186/s12870-022-03469-0

**Published:** 2022-02-22

**Authors:** Ao Li, Ai Liu, Shuang Wu, Kunjing Qu, Hongyin Hu, Jinli Yang, Nawal Shrestha, Jianquan Liu, Guangpeng Ren

**Affiliations:** 1 State Key Laboratory of Grassland Agro-Ecosystems, College of Ecology, Lanzhou University, Lanzhou, China; 2 Key Laboratory of Bio-Resource and Eco-Environment of Ministry of Education & State Key Lab of Hydraulics and Mountain River Engineering, College of Life Sciences, Sichuan University, Chengdu, China

**Keywords:** Chromatin organization, Genome assembly, *Medicago truncatula*, Jemalong A17, R108, Structural variants

## Abstract

**Background:**

Structural variants (SVs) constitute a large proportion of the genomic variation that results in phenotypic variation in plants. However, they are still a largely unexplored feature in most plant genomes. Here, we present the whole-genome landscape of SVs between two model legume *Medicago truncatula* ecotypes–Jemalong A17 and R108– that have been extensively used in various legume biology studies.

**Results:**

To catalogue SVs, we first resolved the previously published R108 genome assembly (R108 v1.0) to chromosome-scale using 124 × Hi-C data, resulting in a high-quality genome assembly. The inter-chromosomal reciprocal translocations between chromosomes 4 and 8 were confirmed by performing syntenic analysis between the two genomes. Combined with the Hi-C data, it appears that these translocation events had a significant effect on chromatin organization. Using both whole-genome and short-read alignments, we identified the genomic landscape of SVs between the two genomes, some of which may account for several phenotypic differences, including their differential responses to aluminum toxicity and iron deficiency, and the development of different anthocyanin leaf markings. We also found extensive SVs within the nodule-specific cysteine-rich gene family which encodes antimicrobial peptides essential for terminal bacteroid differentiation during nitrogen-fixing symbiosis.

**Conclusions:**

Our results provide a near-complete R108 genome assembly and the first genomic landscape of SVs obtained by comparing two *M. truncatula* ecotypes. This may provide valuable genomic resources for the functional and molecular research of legume biology in the future.

**Supplementary Information:**

The online version contains supplementary material available at 10.1186/s12870-022-03469-0.

## Background

DNA structural variants (SVs) > 50 bp in length are a major resource of genomic variation and often have profound consequences on phenotypic variation. Despite this, the impact of SVs, such as presence/absence variants (PAVs), insertions, deletions, translocations, and inversions is an unexplored feature in many plant genomes [1]. SVs benefit plant breeders, drug developers, and other professions that regularly take advantage of natural variation in plant populations [2–5]. Although single nucleotide polymorphisms (SNPs) capture some meaningful genomic variations that contribute to phenotypic differences, SVs account for more heritable nucleotide variations than SNPs do [6, 7]. For example, SVs are three times more likely to be associated with a phenotype than SNP is in humans [7]. In addition, SVs have been associated with human diseases, such as cancer [8] and schizophrenia [9]. In plants, SVs are associated with phenotypic variations such as fruit shape [10], leaf size [11], and fruit color [1]. Therefore, studying SVs is critical for understanding phenotypic diversity in plants [12, 13]. A high-quality reference genome is needed to identify such variations. With the development of high-throughput sequencing technology, it is becoming much easier and cheaper to assemble high-quality reference genomes. Consequently, comprehensive SV catalogs are beginning to appear for some animal and model plants such as songbird [14], rhesus macaque [15], rice [16], and maize [17]. On the other hand, SVs in most plant species, including the model legume plant *Medicago truncatula*, remain unexplored.

*Medicago truncatula* is a model for investigating various aspects of legume biology, particularly on their symbiotic relationships with rhizobia and arbuscular mycorrhizae, organ development, their secondary metabolism, and their responses to biotic and abiotic stresses [18–21] because of its short generation cycle, small genome size, amenability to genetic transformation, and self-fertility. Although *M. truncatula* has many diverse ecotypes, two of them (ecotypes Jemalong A17 and R108, hereafter A17 and R108, respectively) are mainly used for functional genomic studies [22–24]. A17 was originally isolated from Australia and used for a whole-genome sequencing project [25, 26], whereas R108 was derived from ecotype R108-1 through in vitro regeneration and is often used for gene transformation [27]. R108 is phylogenetically distant from A17 [23], and some degree of reproductive isolation exist between them, especially when R108 serves as the female parent during crossing [28]. Several of their phenotypic traits, including anthocyanin leaf markings, tolerance to drought and salt stress, response to mineral toxicity, jasmonic acid/ethylene-induced resistance and nitrogen fixation, are also considerably different [18, 29–32]. However, the genomic basis of these phenotypic differences between the two ecotypes is understudied, partly due to lack of a chromosome-level genome assembly for R108.

Some differences between the A17 and R108 genomes have been identified. When there was no high-quality R108 genome available, previous studies used the A17 genome as a reference for R108 gene mapping. Due to their distant phylogenetic relationship and a large inter-chromosomal rearrangement between chromosomes 4 and 8 [24, 33], these mapping analyses likely produced inaccurate syntenic alignments. A chromosome-scale genome assembly for R108 was produced by Kaur et al*.* [34] using 48 × Hi-C data based on the previously published scaffold-level genome assembly (R108 v1.0), but they did not perform whole-genome comparisons to identify SVs. Zhou et al*.* [35] constructed a pan-genome by mapping 15 de novo* M. truncatula* assemblies to the A17 Mt4.0 reference genome and explored different types of SVs among them. Wang et al*.* [32] identified SVs, indels, SNPs, and found that some SVs are associated with the differential response of A17 and R108 to aluminum and sodium toxicity by mapping R108 resequencing data to an earlier version of the A17 genome assembly (Mt3.5). Yet, information about the whole-genome landscape of SVs and their effects on chromatin organization in A17 and R108 remain unknown [36, 37].

In this study, we first re-assembled a chromosome-scale R108 genome using 124 × Hi-C data [34] and performed genome annotation and evolutionary analyses on this ecotype. We also generated 389 × Hi-C data for A17 to characterize and compare chromatin organization in the euchromatic (compartment A) and heterochromatic (compartment B) regions of the genome, respectively, in the two ecotypes. Next, we performed whole-genome alignment using our high-quality genome assemblies to identify SVs. We compared these with results obtained from short-read data. Finally, we investigated genomic regions (sequences within or near known genes) associated with phenotypic differences between A17 and R108.

## Results

### An improved R108 genome assembly

**Fig. 1:**
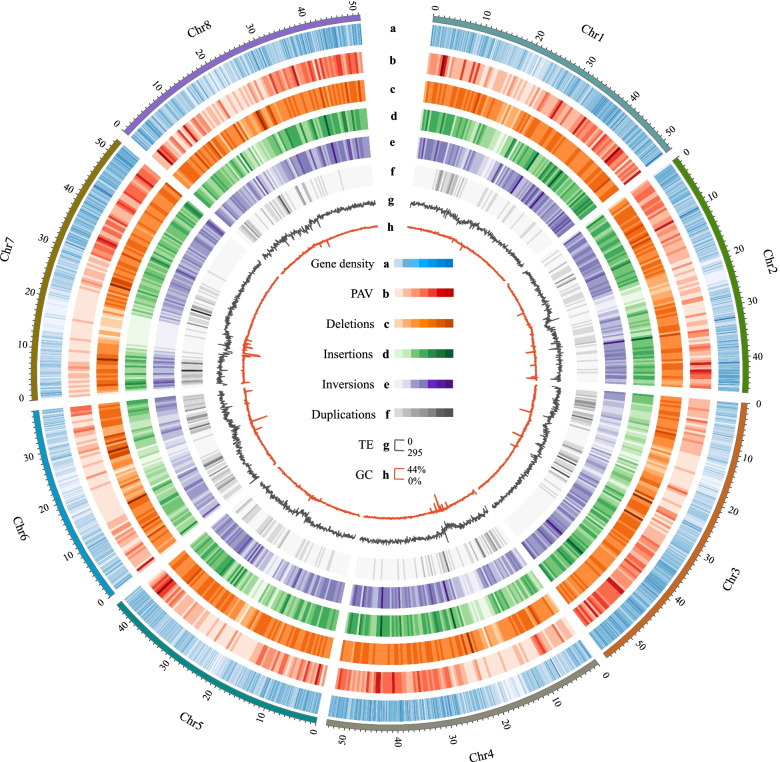
An overview of the genomic features and SV map analyses of the R108 genome. **A** Gene density was calculated in 100 kb windows. **B** The distribution of PAV density in R108 as determined using whole-genome alignment in 500 kb windows. **C**-**F** The distributions of the four types of SVs based on short reads including deletions, insertions, inversions, and duplications in 500 kb windows. **G** Transposable element (TE) content in 100 kb windows. **H** GC content in 100 kb windows

To identify SVs between the genomes of the two *M. truncatula* ecotypes (A17 and R108), we first used Hi-C technology to increase the resolution of the published, scaffold-level R108 v1.0 genome [33] to chromosomal scale. By performing hierarchical clustering on ~ 49 Gb (~ 124 × coverage) of Hi-C data, it was determined that approximately 393 Mb (97.8%) out of the total contig length (402 Mb) were anchored to eight pseudochromosomes (Fig. S1, Tables S1 and S2). This is 3 Mb more than the recently published MedtrR108_hic genome assembly reported (Table S3) [34]. A total of 42,066 protein-coding genes were annotated based on a combination of de novo, homology-based and transcriptome-based predictions, and 97.5% of the total genes were found on chromosomes (Table 1, Table S2). The distribution of gene density and GC content along each chromosome were uneven (Fig. 1). Benchmarking Universal Single-Copy Orthologs (BUSCO) evaluation showed that the gene set completeness of the R108 genome was comparable to that of the A17 genome (Mt5.0), in that more than 98% of all BUSCOs were successfully annotated (Table S4). Five protein databases–InterPro, KEGG, NR, SwissProt, and KOG–were used to evaluate our protein models. Overall, we assigned potential functions to 93.25% (39,225) of the protein-coding genes in the R108 genome (Table S5). In addition, a total of 394 microRNAs (miRNA), 1,298 ribosomal RNAs, 1,163 small nuclear RNAs (snRNA) and 1,162 transfer RNAs (tRNA) were identified in the R108 genome (Table 1).

**Table 1:** Summary of R108 genome assembly and annotation

Categories	Type	Length (bp)	No	% of genome
Assembly [33]	Contigs	399,348,944	1,005	-
	Contig N50	5,925,378	18	-
Non-coding RNAs	miRNA	44,593	394	0.011
	snRNA	132,508	1,163	0.033
	rRNA	379,649	1,298	0.094
	tRNA	87,387	1,162	0.022
Transposable elements	DNA	49,303,718	-	12.26
	LINE	22,617,452	-	5.62
	SINE	4,771,852	-	1.19
	LTR	75,958,551	-	18.89
	RC	9,565,763	-	2.38
	Satellite	3,054,872	-	0.76
	Simple_Repeat	7,944,656	-	1.98
	Unknown	42,481,132	-	10.56
	Low_Complexity	3,896,467	-	0.97
	Total	187,714,868	-	46.68
Gene	Gene loci	-	42,066	-
	Average gene length (bp)	2,451	-	-
	Average CDS length (bp)	1,070	-	-
	Average exon length (bp)	252.40	-	-
	Average exons per gene	-	4.24	-
	Average intron length	426.58	-	-

### Annotation and comparison of the transposable elements (TEs) in the two genomes

TEs are major components within most genomes and have played important roles in driving plant genome evolution [38]. We used de novo prediction and a homology-based search to annotate repeat sequences within the two genomes. We annotated more TEs in the A17 genome (205.5 Mb; 51.1%) than in the R108 genome (187.7 Mb; 46.7%; Tables 1 and S6). This difference was primarily caused by one TE known as Gypsy, which is a long terminal repeat (LTR) retrotransposon (Table S6). The two genomes were reciprocally compared with each other to identify specific TE insertions. A total of 4,459 TE insertions affecting 256 genes were identified in the A17 genome, while 4,346 TE insertions affecting 430 genes were identified in R108 (Table S7). Gene Ontology (GO) enrichment analysis showed that TE-affected genes in R108 were only enriched in nucleotide binding processes, whereas nine GO terms, including activity-related molecular functions and some metabolic processes, were associated with the TE-affected genes in A17 (Table S8).

### Comparative genomic analyses

To study the evolutionary distance between R108 and A17, annotated genes from 11 species of the Leguminosae family (i.e., *Medicago sativa*, *Medicago ruthenica*, *Trifolium pertense*, *Pisum sativum*, *Cicer arietimum*, *Lotus japonicus*, *Glycine max*, *Phaseolus vulgaris*, *Cajanus cajan*, *Arachis duranensis*) and one rosid species (*Arabidopsis thaliana*) were clustered into gene families (Fig. S2). We identified 553 single-copy homologous genes from these 13 genomes for phylogenetic analysis. As expected, R108 displayed the closest phylogenetic relationship with A17. Both diverged from a common ancestor about 1.44 million years ago (Ma, Fig. S2). The phylogenetic relationships among these 13 species were the same as those recovered by previous studies [39, 40]. By comparing the gene families in other plant species (Table S9), we detected 1,347 expanded and 2,701 contracted gene families in R108. A total of 1,721 expanded and 2,254 contracted gene families were identified in the A17 genome (Table S9). The contracted gene families in R108 were mainly enriched in various binding functions and catabolic processes (Table S10). Various ion binding functions such as phosphate ion binding (GO:0,042,301), magnesium ion binding (GO:0,000,287), metal ion binding (GO:0,046,872) and transition metal ion binding (GO:0,046,914) were enriched in the expanded gene families (Table S11).

### Global comparisons and differences in chromatin organization between the R108 and A17 genomes

**Fig. 2:**
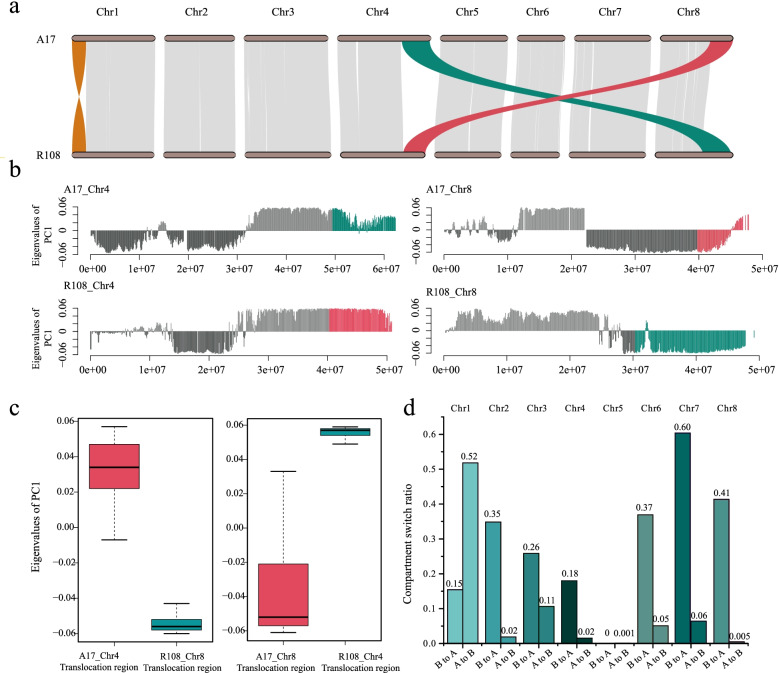
Gene synteny and comparative compartmental analysis between A17 and R108. **A** Gene synteny between the A17 and R108 genomes. **B** Compartment status in chromosomes 4 and 8 of the A17 and R108 genomes. The positive values in the first eigenvector represent compartment A and the negative values represent compartment B. The colors represent inter-chromosomal reciprocal translocations. **C** Boxplot showing the A/B compartment status of the translocation regions. **D** Bar graph showing compartmental status switches (i.e., A to B, and B to A) across the eight chromosomes in R108 using the compartmental status of the corresponding syntenic regions in A17 as a reference

Except for chromosome 8, each chromosome in R108 was shorter than its corresponding chromosome in A17 (Fig. S3). With the improved R108 genome assembly, syntenic analysis was performed. The syntenic blocks revealed high chromosome-to-chromosome collinearity between the two genomes (Fig. 2a, Fig. S4). Among all the syntenic blocks, one-to-one syntenic blocks accounted for 84% (361 Mb) of the A17 genome and 94% (378 Mb) of the R108 genome (Fig. S5). These percentages are much higher compared with those reported by a previous study [33], in which only 280 Mb of syntenic regions in the scaffold-level R108 assembly (R108 v1.0) were recovered (Mt4.0). We also confirmed the inter-chromosomal reciprocal translocation between chromosomes 4 and 8 and found a large inversion on chromosome 1 (Fig. 2a). Based on our improved R108 genome assembly, the size of the inter-chromosomal reciprocal translocation site was comparable to that reported by Kaur et al*.* [34]. However, we identified more syntenic genes in this region (Table S12).

To determine whether genomic rearrangements (such as inversions, translocations, and PAVs) differentially affected chromatin organization between the two genomes, we used HiC data (A17 389 × , R108 124 × ; Table S1) to identify conserved A/B compartments within each genome. Using the genomic compartment of A17 as a reference, we found that 55.19% (217 Mb) of the total length of the syntenic regions in R108 have the same compartment status as A17. On the other hand, 64.92 (16.51%) and 112.27 Mb (28.30%) of the syntenic regions in R108 exhibited A to B and B to A compartmental transitions, respectively (Fig. S6). The longest A to B compartmental switch occurred on chromosome 1 (26.97 Mb), whereas the longest opposite switch was found on chromosome 7 (31.54 Mb, Fig. 2d, Table S13). We found nearly no A/B compartment switches on chromosome 5 by comparing the two genomes (Fig. 2d, Fig. S7), indicating that chromosome 5 was conserved following the divergence of the two ecotypes. The results showed that the inter-chromosomal reciprocal translocation regions between chromosomes 4 and 8 exhibited opposite compartment status (Fig. 2b and c, Fig. S7), meaning that the translocation region on chromosome 4 in A17 is part of the A compartment and its syntenic region on chromosome 8 in R108 is part of the B compartment. The other translocation also exhibited opposite compartment status (*i.e.,* B compartment in A17 vs*.* A compartment in R108). Moreover, A compartments comprised 50.88% and 61.07% of the regions containing PAVs in A17 and R108, respectively (Fig. S8, Table S14), indicating that large-scale insertions were biased towards euchromatic regions.

### SVs between the R108 and A17 genomes

**Fig. 3:**
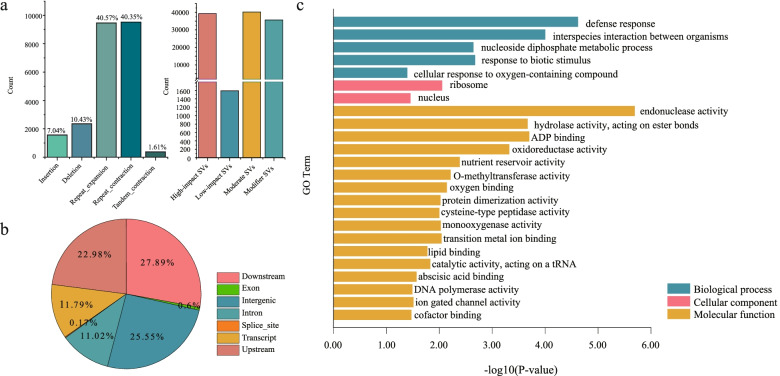
SVs identified based on whole-genome alignment in the R108 genome and SV functional enrichment. **A** Number of SVs in R108, including deletions, insertions, repeat_expansion, repeat_contraction, and tandem_contraction. Also includes the number of genes in each impacted-by-SV category (High-impact, Medium-impact, Low-impact, and Modifier-impact; see Table S16 for detailed definitions). **B** Annotation of all SVs categorized using SnpEff based on their positions in the annotated R108 genome. ‘Downstream’ represents SVs located at least 5 kb downstream from a gene; ‘Intergenic’ indicates SVs in the intergenic regions; ‘Splice_site’ indicates a splice variant that changes the 2 bp region at the 3' or 5' end of an intron; ‘Transcipt’ indicates a feature ablation whereby the deleted region includes a transcript feature; ‘Upstream’ indicates SVs located at least 5 kb upstream of a gene. **C** GO enrichment of the highly-impacted-by-SV genes

After improving the R108 genome assembly, we detected SVs between the two ecotypes using two approaches. We first compared the A17 and R108 primary assemblies using whole-genome alignment to identify SVs in R108. A total of 23,455 R108 genomic SVs were identified, with PAVs only accounting for 17.47% of the SVs, and all the remaining SVs being repeat-mediated (Fig. 3a, Table S15). Most SVs were detected in non-coding regions, while 0.6% of the SVs were present in exon regions, which could have affected gene function and led to phenotypic divergence between the two ecotypes (Fig. 3b). The distribution of the PAVs across the eight chromosomes was uneven (Fig. 1b), indicating that the chromosomes changed more rapidly on the arms than at the center, consistent with previous studies [41]. 18,604 genes were categorized as SV-high-impact genes (i.e., the SV is assumed to have a high or disruptive impact on the protein by causing protein truncation, causing loss of function, or triggering nonsense mediated decay; Fig. 3a, Table S16) which were mainly enriched in “defense response”, “response to biotic stimulus”, “interspecies interaction between organisms”, “transition metal ion binding” and some categories of activity-related molecular functions (Fig. 3c).

Next, we mapped an average of ~ 37 × Illumina reads from 10 R108 individuals to the A17 genome assembly (Mt5.0). This generated 78,634 SVs which included 52,994 deletions, 10,901 insertions, 9,038 duplications, and 5,701 inversions (Fig. S9, Table S17). The number of SVs was significantly higher compared to those identified by mapping R108 genome resequencing data to an earlier version of A17 genome assembly (Mt3.5) [32]. While the two comparisons produced different types of SVs (Fig. 3a, Fig. S9a), we could only compare the PAVs. Although more than 95% of the PAVs inferred by the whole-genome alignment were confirmed by the short-read alignment analysis, many Illumina-based PAVs were not identified during whole-genome alignment analysis (Fig. S10). Genomic variation within the 10 R108 individuals may partly account for this discrepancy. The SVs detected with short reads had a high impact on 39,245 genes (Table S16). The GO enrichment analysis suggested that these highly impacted genes were related to “response to stimulus”-associated biological processes such as “interspecies interaction between organisms”, “cellular response to stimulus” and “response to stimulus and anion transport” (Fig. S9c). It should be noted that the huge difference between whole-genome alignment and short reads in terms of the number of highly impacted genes should be treated with caution, as these highly impacted genes were computationally categorized. Thus, these results should only be used for guidance in future studies.

### SVs in putative genes related to phenotypic variations

Although the two ecotypes have many different phenotypic traits, the relative contribution of SVs to these variations was largely understudied. Previous studies suggested that SVs in the genes (*e.g., MtAACT* and *MtFRD3*) of these two ecotypes may account for their differential responses to mineral nutrient deficiency and mineral toxicity [29, 32]. Previously detected SVs in *MtAACT* and *MtFRD3* were also discovered during our analysis. Furthermore, the genes that were differentially expressed in A17 and R108 under drought stress (*e.g., MtZEP* [42]) and iron deficiency (*e.g., MtASCO1* [32]) also contained SVs (Fig. S11). Wang et al*.* [32] found that the *YSL* gene was deleted in R108 compared to the A17 Mt3.5 assembly, which could account for the lower accumulation of iron in R108 compared to A17. Yet, our results suggested that the copy number of *YSL* was identical between the two ecotypes (Table S18). The large-scale SV resources detected here will provide a foundation for further functional and molecular research between A17 and R108.

**Fig. 4:**
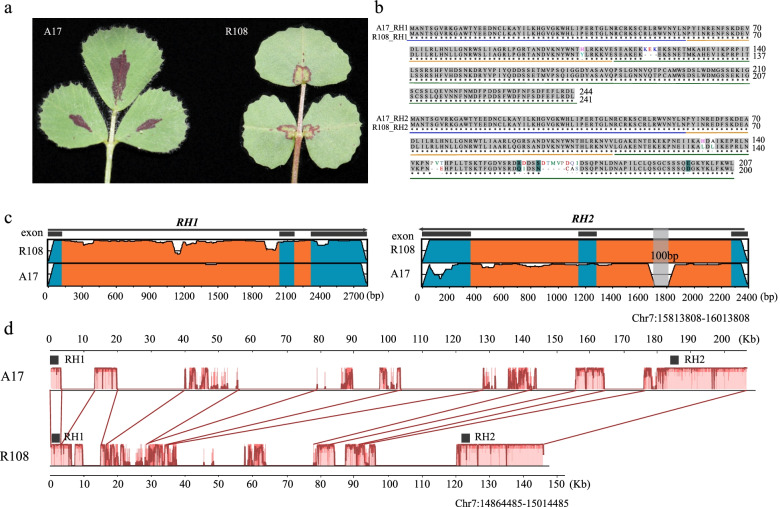
Gene variations within putative genes governing anthocyanin leaf markings in A17 and R108. **A** Differences in the anthocyanin leaf markings between the two ecotypes. **B** Alignment of the protein sequences of the *RH1* and *RH2* genes. Blue and orange underlines indicate the R2 repeat domain (R2 domain) and R3 repeat domain (R3 domain), respectively. The green underline indicates the C-terminal domain (CTD). **C** VISTA sequence conservation plot of the *RH1* and *RH2* genes obtained by comparing A17 to R108. The gray area indicates a 100 bp deletion located within the first intron of *RH2*. **D** Sequence conservation plot of the intergenic regions obtained by comparing the two genes

One of the remarkable morphological differences between A17 and R108 is their anthocyanin leaf markings (Fig. 4a). R108 has a yellow spot with a strong red border on the adaxial surface in the basal part of the leaflets. By contrast, A17 has an enhanced, enlarged red border. A previous study showed that the anthocyanin leaf spot marking on R108 is controlled by two antagonistic MYB paralogs, RED HEART1 (*RH1*) and *RH2* [18], and both were categorized as highly-impacted-by-SV genes based on our analysis. To further understand sequence variation in these genes between A17 and R108, we identified the sequences of the two genes in both ecotypes and compared their gene structures. Sequence alignment showed that *RH1* and *RH2* are highly conserved in both the R2 and R3 domain regions of A17 and R108, respectively. Major differences exist within their C-terminal domains (CTD, Fig. 4b, Fig. S12). In the CTD of *RH1*, there were three amino acid deletions in R108 that were not present in A17. Even more variations were observed in the CTD of *RH2* (Fig. 4b). A previous study suggested that CTD divergence results in the sub-functionalization of *RH1* and *RH2*. Variation within the CTD of each gene could have also played a role in the formation of different anthocyanin leaf markings in A17 and R108. In addition, large SVs were found in the first intron of *RH2* and the intergenic region between *RH1* and *RH2* (Fig. 4c and d). These SVs and the sequence variation in the CTD region may contribute to differences in the anthocyanin leaf markings in the two ecotypes.

### SVs in putative genes related to nitrogen-fixing symbiosis

**Fig. 5:**
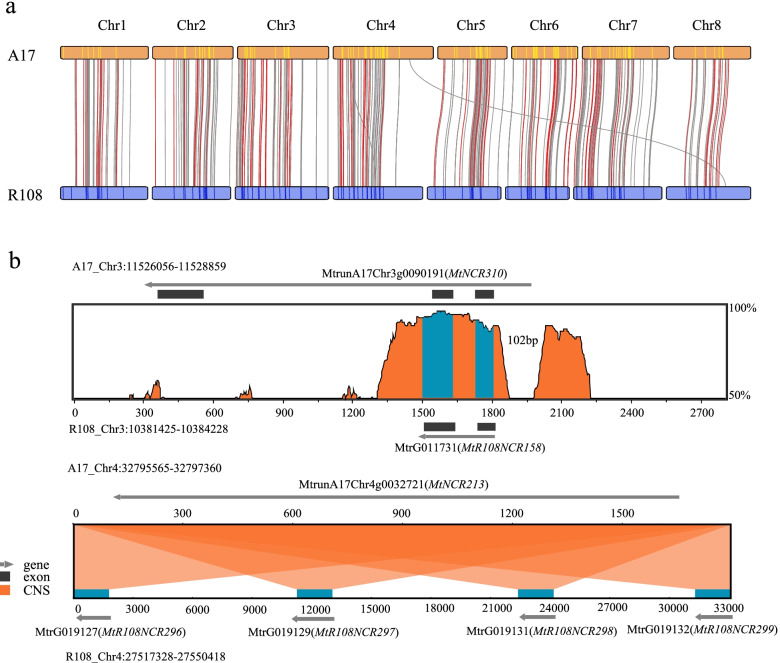
SVs between A17 and R108 in nitrogen-fixing symbiosis-related (NCR) genes. **A** Chromosomal locations of NCR genes in R108 and their syntenic regions in A17. The gray (without SVs) and red (with SVs) lines indicate syntenic relationships of the NCR genes between the two genomes; The yellow lines indicate NCR genes only found in A17; The blue lines indicate NCR genes only found in R108. **B** Two examples of SVs between A17 and R108 within NCR genes visualized using a VISTA sequence conservation plot

Interestingly, we found that the GO enrichment analyses done on highly-impacted-by-SV genes identified using whole-genome and short reads were both related to the GO term “interspecies interaction between organisms” (Fig. 3c, Fig. S9c). Most genes related to this GO term belonged to the nodule-specific cysteine-rich (NCR) family, which is only present in the inverted repeat lacking clade (IRLC) of legumes. These genes regulate bacteroid differentiation and activity as positive regulators of effective symbiosis [43, 44]. Only 20 NCR genes in R108 were identified using our gene annotation method, while 678 NCR genes were identified in A17 Mt5.0. Since NCR genes are small secretory peptides (SPPs), most SPPs may have not been identified by our pipeline, as it was intended for gene discovery. To more effectively search for NCR genes in R108, we used the 678 NCR genes in A17 as a query and combined the result with SPADA software (Small Peptide Alignment Discovery Application), which is proven to efficiently identify SPPs [45]. A total of 616 putative NCR genes were identified in the R108 genome. The NCR genes had similar distribution patterns along the chromosomes of the two genomes (Fig. S13, Table S19), and 495 R108 NCRs were syntenic with A17 (Fig. 5a, Table S20), reflecting the recent divergence of the two ecotypes.

We then investigated the chromatin compartmental status of the NCRs in both genomes. The results suggested that 370 (60.06%) out of the 616 NCRs in R108 are in the A compartment and 239 (38.8%) are in the B compartment. In A17, by contrast, more NCRs (382, 56.34%) were in B compartment than in compartment A (281, 41.44%) (Table S19; Table S20). Of the 495 syntenic NCRs, 332 (67.07%) had the same compartment status (Table S20).

For the 495 syntenic NCR genes (including 2 kb of flanking regions), we further investigated the two genomes for the presence of SVs and TE variations. We found that 336 (67.9%) of them were affected by SVs. Of these, 164 were affected by PAVs and 172 were affected by repeat-mediated SVs (Table S20). For example, the gene *MtrG011731* in R108 that was otherwise syntenic with the NCR gene *MtNCR310* in A17 lacked the third exon sequence. Four tandemly duplicated NCR genes in R108 (*MtrG019127*, *MtrG019129*, *MtrG019131*, and *MtrG019132*) were all syntenic with one NCR gene (*MtNCR213*) in A17 (Fig. 5b). We also found that all NCR genes and their 2 kb flanking regions in both genomes contained TEs, although the number of TEs in/near each gene varied (Table S19; Table S20). Two-hundred and eighty-two out of the 336 SV-affected syntenic NCR genes co-occurred with TEs, accounting for 83.93% of the total SV-affected NCRs (Table S20). Extensive differences in the compartmental status, SV landscapes, and TE variability in/near the NCR genes between the two genomes could affect effective symbiosis, although this claim needs further investigation.

## Discussion

*Medicago truncatula* ecotype R108 is widely used in functional genomic studies because of its short generation cycle, small genome size, self-fertility, and most importantly, its much higher transformation efficiency than A17. Compared to A17, though, whose genome assembly has been improved several times, only one chromosome-scale genome assembly exists for R108, and this was published very recently [34]. This paucity of information inhibits our understanding the genomic evolutionary history and genetic code underlying the molecular biology of this model species. Here, we improved the first assembly of R108 [33] by using more Hi-C data (124 ×) than Kaur et al*.* (48 ×) [34]. The quality of our newly assembled R108 genome is slightly higher than the one generated by Kaur et al*.* [34], with more annotated protein-coding genes and a higher BUSCO value (Table S3). By visualizing syntenic regions between A17 and R108, a high degree of collinearity and inter-chromosomal reciprocal translocation between chromosomes 4 and 8 were confirmed. In addition, we detected a large inversion on chromosome 1. The translocation between chromosomes 4 and 8 and the large inversion on chromosome 1 (Fig. 2a) have also been observed when A17 was compared to *M. truncatula* ecotype A20 [46], *Medicago sativa* [47], and *Medicago ruthenica* [40], indicating that the inter-chromosomal rearrangements and inversion occurred specifically in A17.

Chromatin organization plays an important role in the regulation of gene expression. Previous studies focusing on single organisms have often demonstrated correlations among chromatin interactions, transcriptional activities, and epigenetic modifications (*e.g.* [48]*,*). By comparing the genome-wide chromatin interactions and organizational patterns of closely related pairs of crop species (*e.g.,* cotton and *Brassica*) or model species (*e.g., Arabidopsis* and poplar), recent studies have identified changes in the 3D organization of their genomes, as well as the potential role these changes play in evolutionary and/or phenotypic divergence [37, 49–51]. In this study, we compared chromatin organization in A17 and R108, but only focused on the A/B compartments because the resolution of our HiC data was low. We found that chromatin organization between the two ecotypes was substantially less conserved (66%; Fig. 2d) than that of mammalian and cultivated crop species [50, 52], as well as slightly lower than that of two closely related poplar species (71.52%) [37]. Furthermore, our results suggest that SVs affected chromatin organization in the two ecotypes. For example, the syntenic translocation regions on chromosomes 4 and 8 exhibited opposite compartment status (Fig. 2b). The low level of conservation of chromatin organization and the SV-affected chromatin status change could result in different epigenetic modifications and gene transcriptional activity in the two ecotypes (*e.g.* [37]*,*), thereby contributing to their phenotypic and adaptive divergence. Further studies integrating high resolution HiC data with gene expression and DNA methylation should be conducted to investigate the evolution of chromatin organization and transcriptional regulation during the divergence of the two ecotypes.

SV constitutes a large proportion of genetic variation in the genomes of eukaryotic organisms and can affect the fitness of the organism [53]. Our analyses of SVs in individual and bulked R108 genomes provide an overview of the genomic landscape of SVs between A17 and R108. The highly-impacted-by-SV genes are enriched in many important biological processes and molecular functions such as “defense response”, “oxidoreductase activity” and “ADP binding” (Fig. 3, Fig. S9), which highlights their importance in phenotypic divergence. For example, A17 and R108 differ in their responses to aluminum toxicity. This may be associated with the partial deletion (316 bp) of the second intron of the *MtAACT* gene (a putative aluminum-activated citrate transporter) [32]. Similarly, the high sensitivity of R108 to iron deficiency relative to A17 may be related to a deletion in the intronic regions of *MtFRD3* (a gene responsible for loading iron chelator citrate into xylem) and *MtACO1* [29]. A 370 bp deletion in the intronic regions of *MtZEP* (which may lead to the increase of ABA content and subsequent activation of drought-stress-regulated gene expression) may be responsible for the enhanced drought tolerance of A17 relative to R108 [42]. In all cases, the SVs (*i.e.,* deletion/insertion of intronic sequences) could lead to different levels of gene expression in the two ecotypes [29, 32, 42], resulting in phenotypic divergences.

Our further analysis of the sequence variation of *RH1* and *RH2* genes suggests that SVs may also affect the formation of different anthocyanin leaf markings in A17 and R108 (Fig. 4). These two genes have been suggested to function antagonistically during the formation of anthocyanin leaf markings in R108 [18], in which RH1 is the central regulator and RH2 serves as a molecular rheostat to modulate *RH1*-mediated restricted anthocyanin pigmentation. We found a 100 bp insertion in the first intron of *RH2* in R108 and several large SVs (~ 10–50 kb) in the intergenic region between the two genes (Fig. 4c and d). Disruption of *RH2* leads to an enhanced red spot in R108 that, visually, resembles the anthocyanin leaf markings on A17 (Fig. 4a, [18]), implying that RH2 may have no function in A17 or may function differently in the two ecotypes. The roles of these two genes in anthocyanin leaf marking formation in A17 and the contribution of SVs to phenotypic divergence merit further study.

More interestingly, there were extensive differences in the compartmental status, SV content, and TE variations among the NCR genes in the two genomes (Fig. 5, Table S20). NCR genes are specifically found IRLC legumes that produce indeterminate nodules with a persistent meristem [43]. They are antimicrobial peptides essential for terminal bacteroid differentiation during nitrogen-fixing symbiosis [44, 54]. However, the number, size, and composition of NCR genes varies significantly among the IRLC legumes (7 to ~ 700), which has a direct impact on the morphotype of their bacterial partners [55]. In the A17 genome (Mt5.0), there are 678 annotated NCR genes [25]. This number is comparable to the number of genes identified in the R108 genome (616 NCR genes) during this study. Despite this, many NCR genes were not orthologous between A17 (183 NCR genes) and R108 (121 NCR genes). One hypothesis suggests that, although NCR genes have a single origin, they evolved separately in individual lineages, resulting in lineage-specific NCRs that lack orthologs [44, 55–57]. Orthologs can be found amongst closely related species, but local gene duplications followed by diversification during the expansion of this gene family in *M. truncatula* likely account for the variations in NCR genes that were observed in these two ecotypes. Furthermore, even though 495 NCR genes were orthologous between the two genomes, extensive differences in compartmental status, SV content, and TE contents were detected between these orthologs. Although most NCR genes are highly expressed during nodule organogenesis and are essential for symbiosis [44, 58], only a few genes (*e.g., NCR169, NCR211, NCR247,* and *NCR055*) [20, 55, 59] have been functionally characterized to date. The functions of most NCR genes are still largely unknown. A previous study found that some TEs in the vicinity (< 2 kb) of NCR genes are transcriptionally activated during nodule development [60]. However, very few studies have been conducted to investigate the relative contributions of SVs and TEs within NCR genes to symbiosis in different ecotypes. Our results provide new genetic resources for the future functional characterization of NCR genes.

## Conclusions

In this study, we generated Hi-C data for two *Medicago truncatula* ecotypes (A17 and R108). We also resolved the published R108 genome assembly (v1.0) to chromosomal-scale and characterized its chromatin organization. The results suggest that more than 44% of the syntenic regions between the two genomes underwent compartmental transitions. This was especially prominent in the large, inter-chromosomal translocation between chromosomes 4 and 8, indicating chromatin organization was not well conserved. This could have contributed to the phenotypic divergence of the two ecotypes. The whole-genome landscapes of SVs between A17 and R108 provides valuable genomic evidence for the continued investigation of the genetic mechanisms controlling various phenotypic traits in *M. truncatula* such as their remarkably different anthocyanin leaf markings. We found extensive SV and TE variation within the NCR genes in the two genomes. However, the contribution of these SVs and TEs to effective symbiosis are still unknown. Further studies involving more ecotypes/species, genomic sequencing technologies *(e.g.,* strand-specific RNA-seq, and ribosome profiling), and functional experiments should be employed to better understand the functions of NCR genes.

## Methods

### Plant materials and Hi-C sequencing

We collected young, fresh leaves from the two *M. truncatula* ecotypes (Jemalong A17 and R108) for use in Hi-C sequencing. Seeds of both ecotypes were formally identified and kindly provided by the Noble Research Institute, Ardmore, OK, USA. The seeds were first treated with concentrated sulfuric acid for 5 min, then rinsed thoroughly with water. After chilling at 4℃ for 2 days, the seeds were put in moist Petri dishes to germinate at 25℃ until the radicals were approximately 2 cm. Then seeds were sown into the soil and grown in the greenhouse under the following controlled conditions: 24 °C day/22 °C night temperatures with a 16 h-day/8 h-night photoperiod and 60 to 70% relative humidity. Young leaves from two-week-old seedlings were collected from each ecotype to create the Hi-C libraries. For each library, the chromatin was fixed with formaldehyde in the nucleus, and the cross-linked DNA was digested using the restriction enzyme MboI. The sticky ends of these digested fragments were biotinylated and re-ligated to form chimeric circles. The ligated DNA was sheared into 200–300 bp fragments and the Hi-C libraries were sequenced using the Illumina HiSeq platform. The voucher specimens for the two ecotypes (LA-A17-1 and LA-R108-1) used for Hi-C sequencing were deposited in Lanzhou University, China.

### Chromosome-scale assembly of the R108 genome and identification of genomic compartments in both genomes using Hi-C data

A draft assembly of R108 (v1.0, scaffold-scale, BioProject accession number: PRJNA368719) was downloaded from NCBI. The Hi-C data from R108 was first used to connect the scaffolds to the eight chromosomes. The clean Hi-C data were mapped to the draft genome using bwa v. 0.7.17 [61]. Uniquely mapped Hi-C data were retained, clustered, ordered, and placed onto the eight pseudochromosomes using LACHESIS [62]. A heat map depicting the interaction matrix of the pseudochromosomes was plotted with a resolution of 100 kb. The Hi-C data from both ecotypes were also used to identify compartment regions in the chromosomes using HiC-Pro [63]. For each chromosome, we associated the positive eigenvalues of the first eigenvector (PC1) with the A compartment and the negative values with the B compartment.

### Gene prediction and annotation

Repetitive sequences, including tandem repeats and interspersed repeats (mostly TEs), were identified in the R108 genome. Tandem repeats were annotated using TRF v. 4.09 [64]. TEs were identified using a combination of homology-based and de novo approaches at both the protein and DNA level. At the DNA level, we first used RepeatMasker v. 4.0.7 [65] to search for similar transposable elements based on known repeats in the Repbase database v. 20,181,026 [66]. Then, the RepeatModeler v. 1.0.11 package within RepeatMasker was used to build a de novo repeat database which comprised a repeat consensus database with classification information. Finally, RepeatMasker was used to identify transposable elements using the de novo repeat database. At the protein level, the RepeatProteinMasker function within the RepeatMasker package searched for repeats based on the transposable element protein database. For this step, the WU-BLASTX engine was used.

Three methods were used to predict protein-coding genes: de novo predictions, homology-based predictions, and transcriptome-based predictions. Augustus v. 3.3.2 [67], GlimmerHMM v. 3.0.4 [68], Geneid v. 1.4.5 [69] and Genscan [70] software were used to make de novo predictions. For homology-based predictions, protein sequences from *A. thaliana*, *G. max*, *M. truncatula*, *P. vulgaris*, *P. sativum*, *T. pratense* were downloaded and aligned to the genome assembly using TBLASTN and a cutoff e value of 1e-5. The homologous genomic sequences were aligned against the matching proteins using GeneWise v. 2.4.1 [71] for accurate splice alignments. We used publicly available RNA-seq data from R108 (NCBI accession number: SRP077692) for transcriptome-based predictions. RNA-seq reads were assembled into transcripts using Trinity v. 2.1.1 [72] with default parameters. Ultimately, gene model evidence obtained from the de novo, homolog-based and transcript-based predictions were integrated using EvidenceModeler (EVM, v1.1.1) [73], resulting in a non-redundant consensus gene set. The completeness of the gene set was assessed using BUSCO genes from the embryophyta_odb10 lineage dataset. For non-coding RNA (ncRNA) annotation, tRNA genes were identified using tRNA scan-SE [74] with eukaryote parameters. BLAST [75] was used to search the R108 genome assembly for rRNA sequences with default parameters. miRNA and snRNA sequences were identified based on covariance models deposited in the Rfam [76] database (release 13.0) using INFERNAL [77] software. BUSCO [78] genes in the embryophyta_odb10 data (n = 1,375) were used to assess the completeness and accuracy of the assembled R108 genome. Gene functions were annotated by performing BLAST (e value≦1e-5) searches against four protein databases, *i.e.,* KEGG, NR, KOG and SwissProt. Uniprot and GO annotations were assigned to each protein based on the results of alignment. InterProScan v. 5.0 [79] was used to annotate the functions of the protein-coding genes.

### Identification of genome-wide TE insertions

We used the same pipeline used for the R108 genome to annotate repetitive elements in the most updated A17 genome (Mt5.0; https://medicago.toulouse.inra.fr/MtrunA17r5.0-ANR/). The two genomes were reciprocally compared with each other to identify specific TE insertions in the A17 and R108 genomes. Genome comparisons were performed using nucmer from the MUMmer package [80] with the -mum and -noextend parameters. We defined insertions as a gap length of > 1000 bp in the query genome and < 100 bp in the reference genome. If > 80% of the inserted regions in the query genome were annotated as TE sequences, the insertion was defined as a TE insertion and the corresponding alignment gap in the reference genome was defined as a TE insertion site. If a TE insertion site was in a genic region or a 500 bp flanking genic region, we defined such genes as TE-affected genes in the query genome. GO enrichment analysis was performed on TE-affected genes using TBtools.

### Gene families and phylogenetic analysis

We used OrthoFinder v. 2.2.7 [81] to identify the orthologous groups among 11 Leguminosae species (*M. sativa* (PI464715), *M. ruthenica, T. pertense*, *P. sativum*, *C. arietimum*, *L. japonicus*, *G. max*, *P. vulgaris, C. cajan*, *A. duranensis*), and one rosid species (*A. thaliana*). Single-copy orthologous genes were then extracted from the orthologous clustering results. The contracted and expanded gene families in the 12 species were identified using CAFE v. 3.0 [82] and subjected to GO enrichment analysis. For phylogenetic analysis, we first used MAFFT to perform multiple sequence alignment on the protein sequences of single-copy orthologous genes. Then, the protein sequence alignments were converted into codon alignments. Second, regions with large differences or poor alignment scores were deleted using Gblocks v. 0.91 [83]. Finally, we connected the codon alignment results of all the single copy orthologs to form a supergene for phylogenetic analysis. The phylogenetic tree was reconstructed using RAxML v. 8.2.0 [84]. r8s v. 1.81 [85] was used to calculate the average substitution rate along each branch and the time of species divergence.

### Genome comparison and identification of SVs

Syntenic blocks between the A17 and R108 genomes were identified using MCScanX with default parameters [86]. Whole-genome comparisons were performed between the two genomes using the nucmer (nucmer -maxmatch -l 100 -c 500) function from the MUMmer package. Assemblytics [87] were used to call SVs based on the output of nucmer. To predict the effects of the SVs, a custom Python script [88] was used to reformat the results from Assemblytics, and the effects were annotated using SnpEff [89]. The SnpEff results were classified based on the size and impact of the SVs on gene function. These classifications included high, moderate, low, and modifier. The genes categorized as highly-impacted-by-SV genes were subjected to GO enrichment analysis.

We also used Illumina short reads to identify SVs in R108. Illumina reads from 10 R108 individuals were obtained on a HiSeqTM2000 sequencing platform in 150 pair-end (PE) mode. We mapped the Illumina PE reads of the 10 individuals which corresponded to ~ 37 × coverage of the A17 reference genome reference. The BWA-MEM v.0.7.17 module was used to perform the alignment [90]. The resulting bam files were purged of PCR duplicates [91]. SVs were then called using all the samples with LUMPY v.0.2.13 [92] and DELLY v.0.7.7 software [93]. The SV calls from LUMPY and DELLY were merged using SURVIVOR v.1.0.3 [94]. We only retained the SVs that met the following three criteria: (1) had a minimum of three PE reads or split reads supporting the given SV event across all 10 samples; (2) had a minimum SV length of 50 bp and (3) passed the quality filters recommended by LUMPY and DELLY (flag PASS). The effects of these filtered SVs were evaluated as described above, and the highly-impacted-by-SV genes were subjected to GO enrichment analysis.

### Identification of NCR genes and some genes associated with phenotypic divergence

We performed SPADA [45] and BLASTn analyses on all 678 NCR genes in A17 as a query to identify NCR genes in the R108 genome. The peptide sequences were searched using the HMM profile of the Nodulin_late domain (Pfam no. PF07127) available in an hmmscan subprocess of HMMER 3.2.1 (http://hmmer.org/). We merged all hits obtained from both analyses and removed the redundant hits. The locations of all identified NCR genes were marked on the eight chromosomes using MapChart v. 2.32 software [95]. To identify putative genes that may be involved in phenotypic divergence, we searched their sequences using the BLASTp homology search tool. All methods used above were performed in accordance with the relevant guidelines and regulations [96].

## Supplementary Information

**Additional file 1.**

**Additional file 2.**

## Data Availability

The Hi-C data from A17 and R108 and the genome resequencing reads from the 10 R108 samples used in this study have been deposited in the NCBI database under the BioProject ID PRJNA771923. The final assembled genome has been deposited in the National Genomics Data Center (https://bigd.big.ac.cn/?lang=en) under the accession number GWHBFSB00000000. The genome annotation information is available at https://github.com/liao20182018/medicago-truncatula-R108-genome. Lists of SVs detected in A17 and R108 based on whole-genome alignment and R108 genome resequencing data are available in the Dryad repository (https://doi.org/10.5061/dryad.bzkh189b7).

## Abbreviations

*M.*: *Medicago*
*T.*: *Trifolium*
*P.*: *Pisum*
*C.*: *Cicer*
*L.*: *Lotus*
*G.*: *Glycine*
*P.*: *Phaseolus*
*C.*: *Cajanus*
*A. duranensis*: *Arachis duranensis*
*A.thaliana*: *Arabidopsis thaliana*
SV: Structural variant
SNP: Single nucleotide polymorphisms
Hi-C: High-throughput chromosome conformation capture
BUSCO: Benchmarking Universal Single-Copy Orthologs
TE: Transposable element
LTR: Long Terminal Repeat
snRNA: Small nuclear RNA
tRNA: Transfer RNA
miRNA: MicroRNA
rRNA: Ribosomal RNA
CDS: Coding Sequence
GO: Gene Ontology
KEGG: Kyoto Encyclopedia of Genes and Genomes
NR: RefSeq non-redundant proteins
KOG: Clusters of orthologous groups for eukaryotic complete genomes
MYA: Million Year Ago
DEL: Deletions
INS: Insertions
DUP: Duplications
INV: Inversions
CTD: C-terminal domain
IRLC: Inverted repeat lacking clade
NCR: Nodule-specific cysteine-rich
CNS: Conserved non-coding sequences
SPADA: Small Peptide Alignment Discovery Application
