# Supplementary material for: Comparison of structural variants in the whole genome sequences of two Medicago truncatula ecotypes: Jemalong A17 and R108
Source: BMC Plant Biol. 2022 Feb 22;22:77. doi: 10.1186/s12870-022-03469-0 (PMC8862580; doi:10.1186/s12870-022-03469-0)
Supplement: Supplementary file 1 — Additional file 1. [file 12870_2022_3469_MOESM1_ESM.doc]

**Supplementary information**


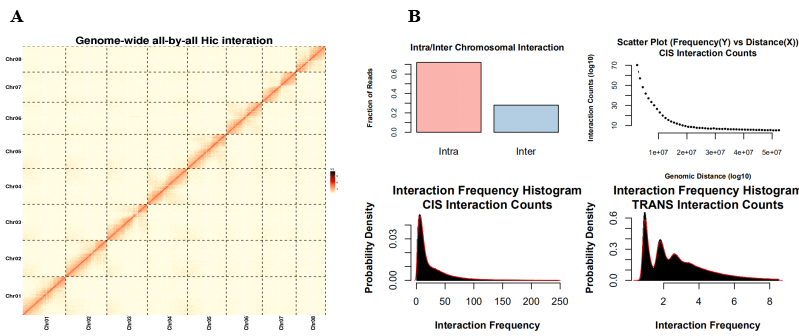
**Figure S1.** Genome-wide all-by-all Hi-C interactions and quality control of the Hi-C data for R108. (A) Genome-wide all-by-all Hic interaction. (B) From top-left to bottom-right: proportion of intra/inter-chromosomal interactions, scatter-plot of interaction counts versus genomic distance between two loci, histogram of interaction counts for intra (CIS) and inter (TRANS) interactions, histogram of distances between two intrachromosomal loci.


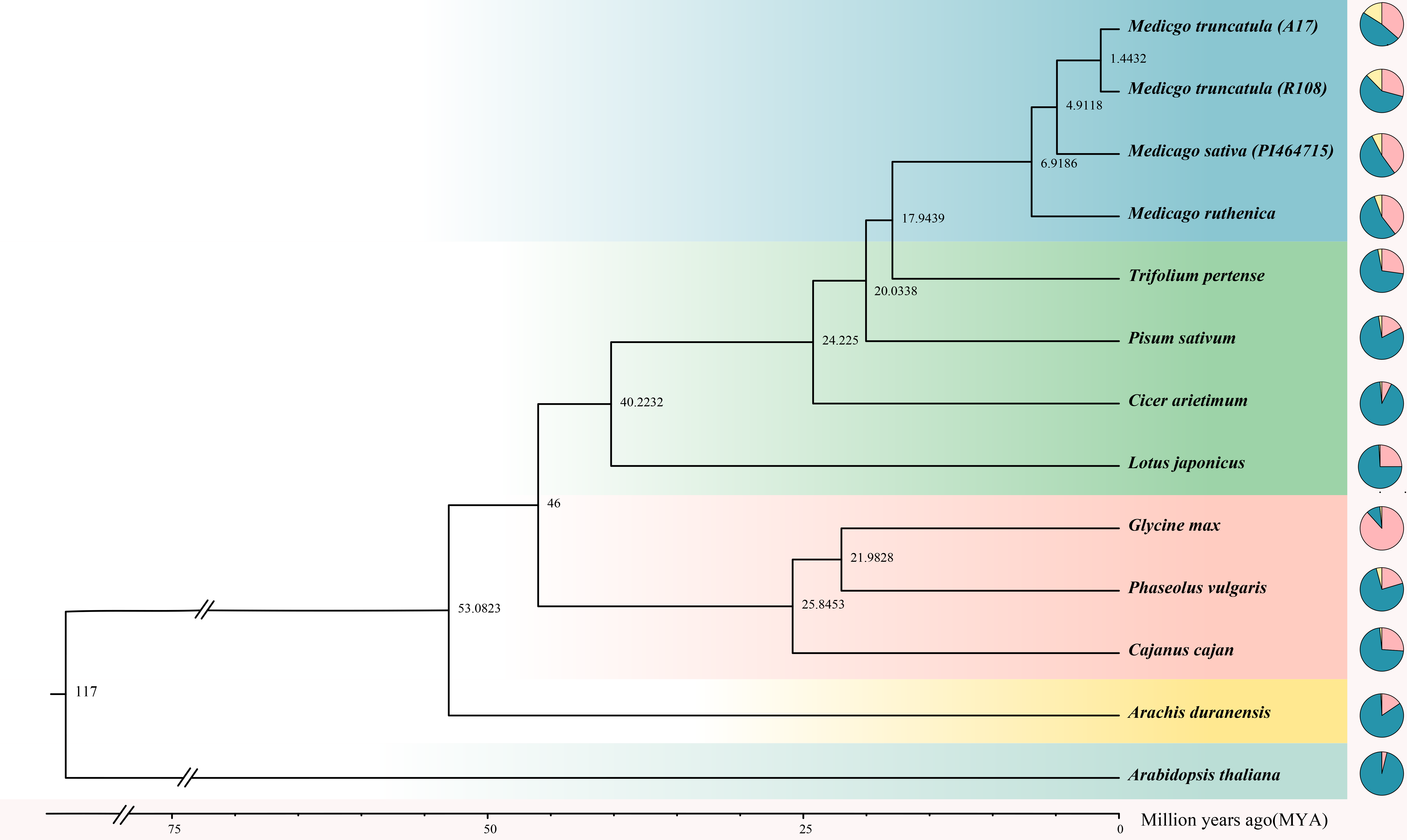
**Figure S2.** Phylogenetic tree of 13 plant species and the evolution of gene families.

The black numerical value beside each node shows the estimated divergence time of each node (MYA, million years ago). Pie chart representation of the numbers of gene-family expansion, contraction, and significant (*p*-value≤0.01) expanded and contracted events are indicated by pink, blue and yellow colores, respectively.


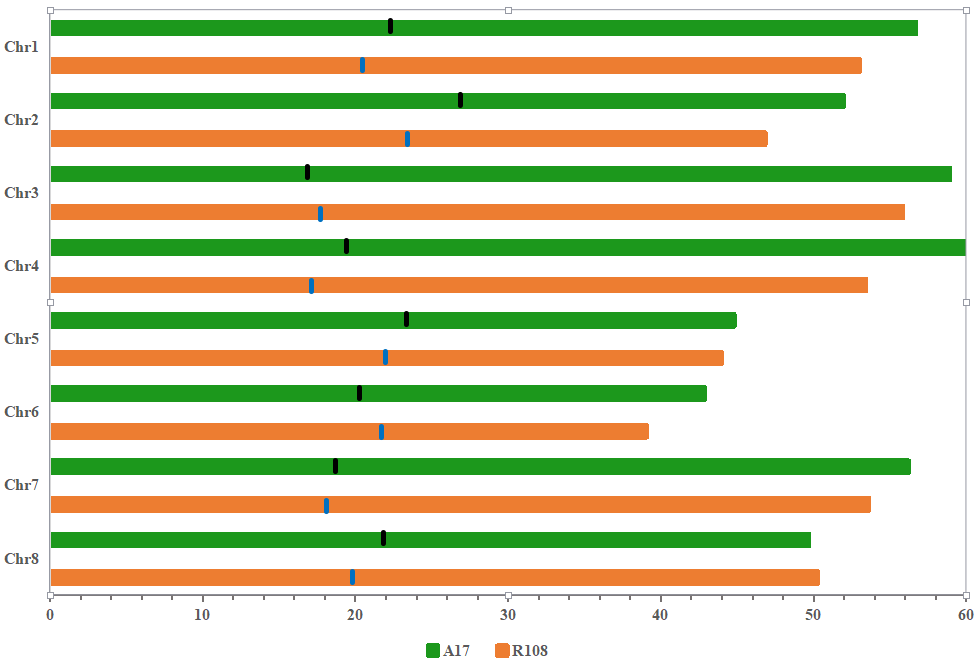
**Figure S3.** Whole-genome comparison of the R108 and A17 genomes.


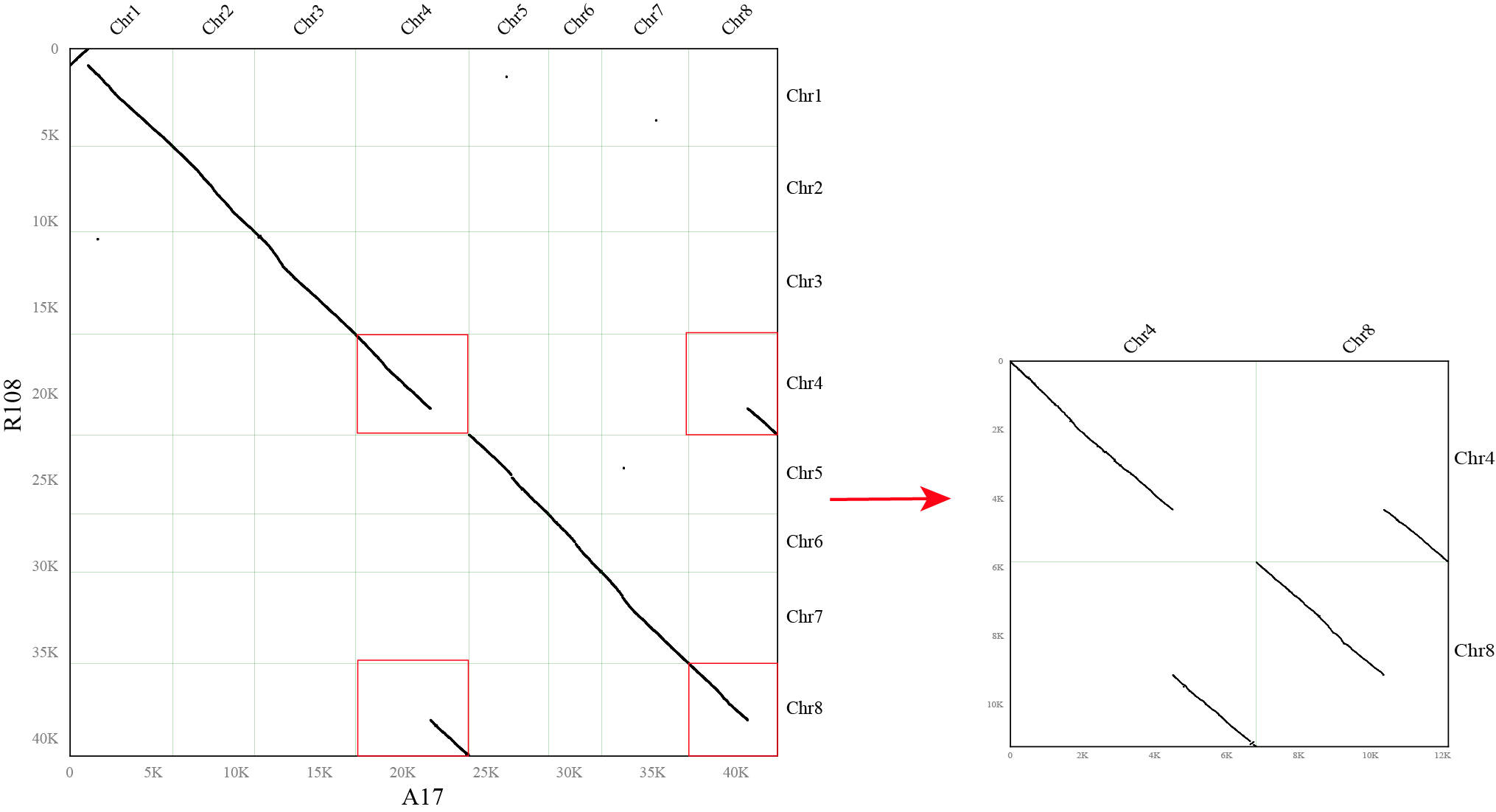
**Figure S4.** Dot plot showing high chromosome-to-chromosome collinearity. The plots show the inter-chromosomal reciprocal translocation between chromosomes 4 and 8 and a large inversion on chromosome 1 between the A17 and R108 genomes.


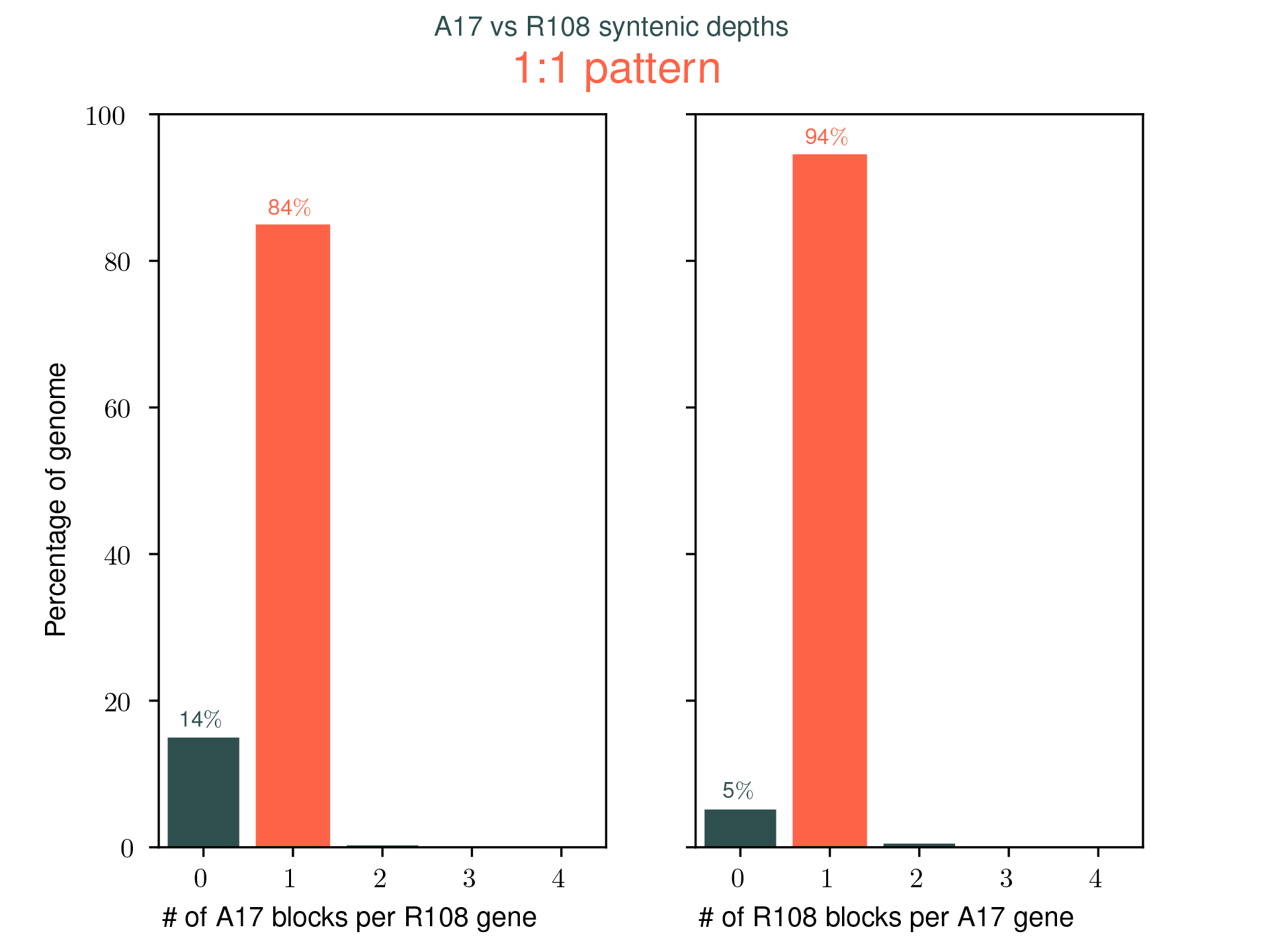
**Figure S5.** Syntenic depths of the A17 and R108 genomes.


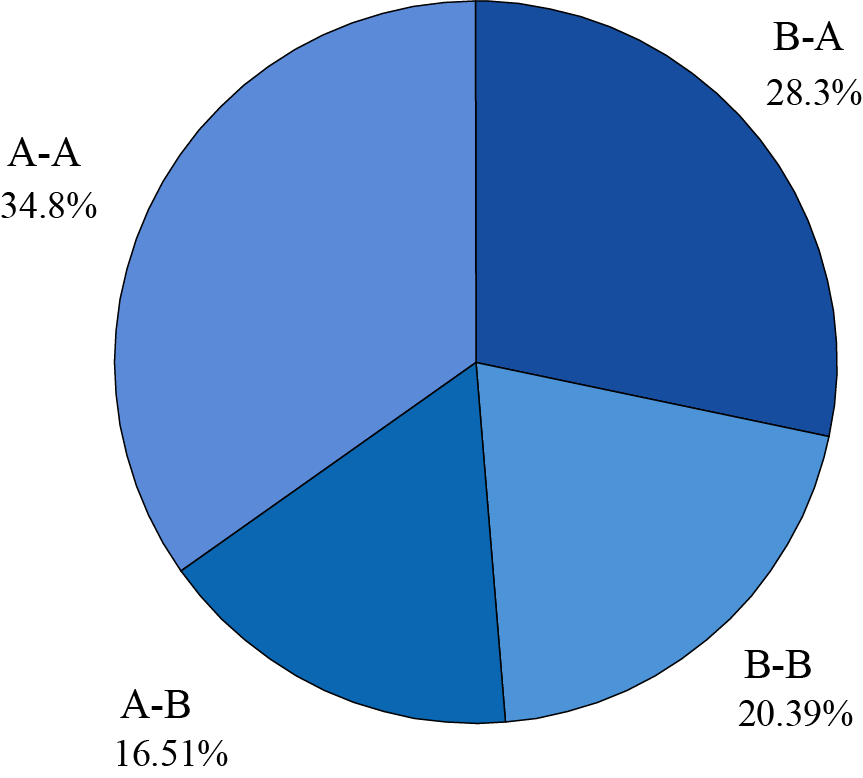
**Figure S6.** Comparison of the compartment status in R108 using A17 as a reference.


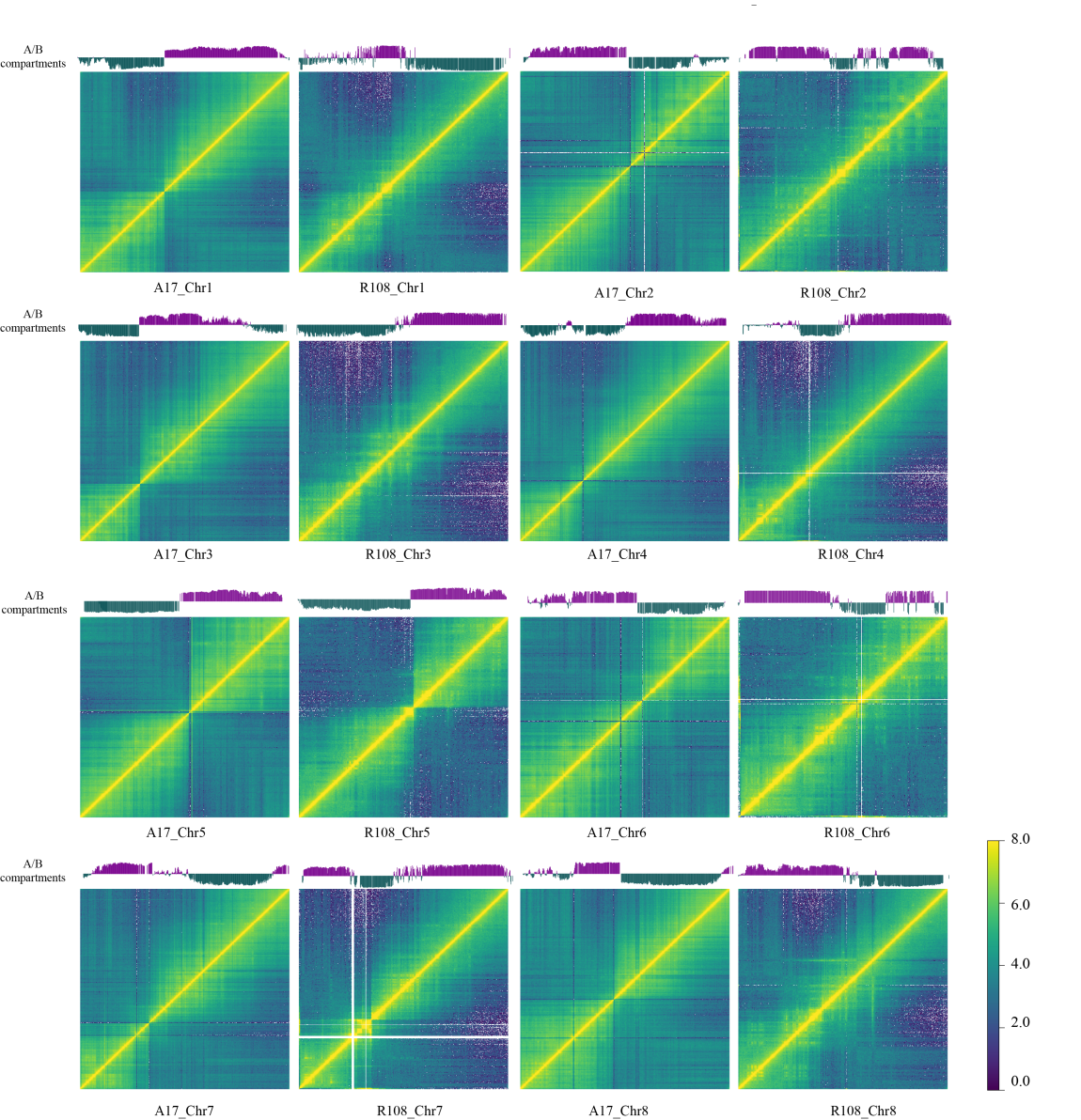
**FigureS7.** Hi-C heatmaps of the A17 and R108 genomes with compartment region results at 100-kb resolution.

**Figure S8.** Analysis of the compartments of the
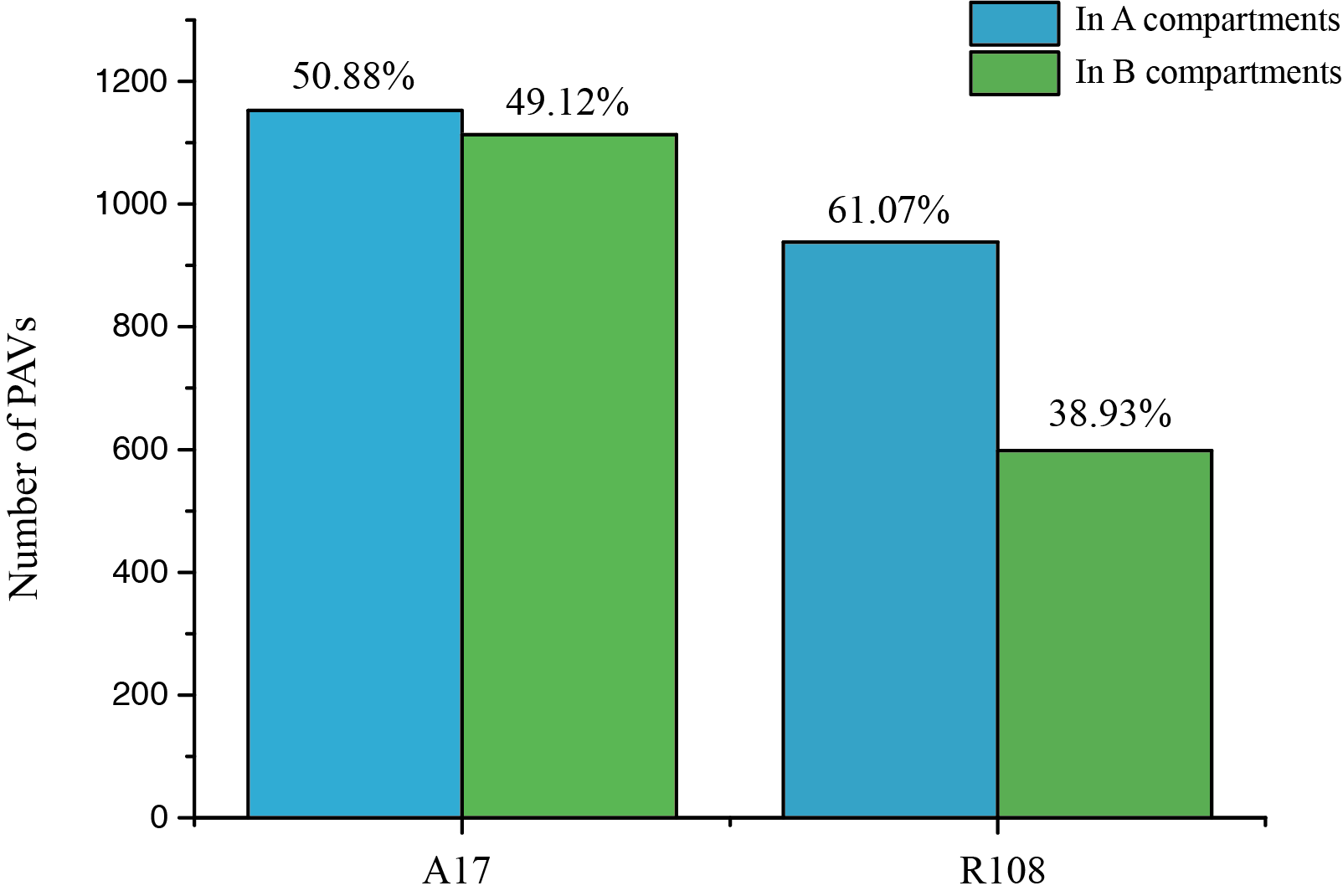
PAVs regions in A17 (left) and R108 (right).

**Figure**
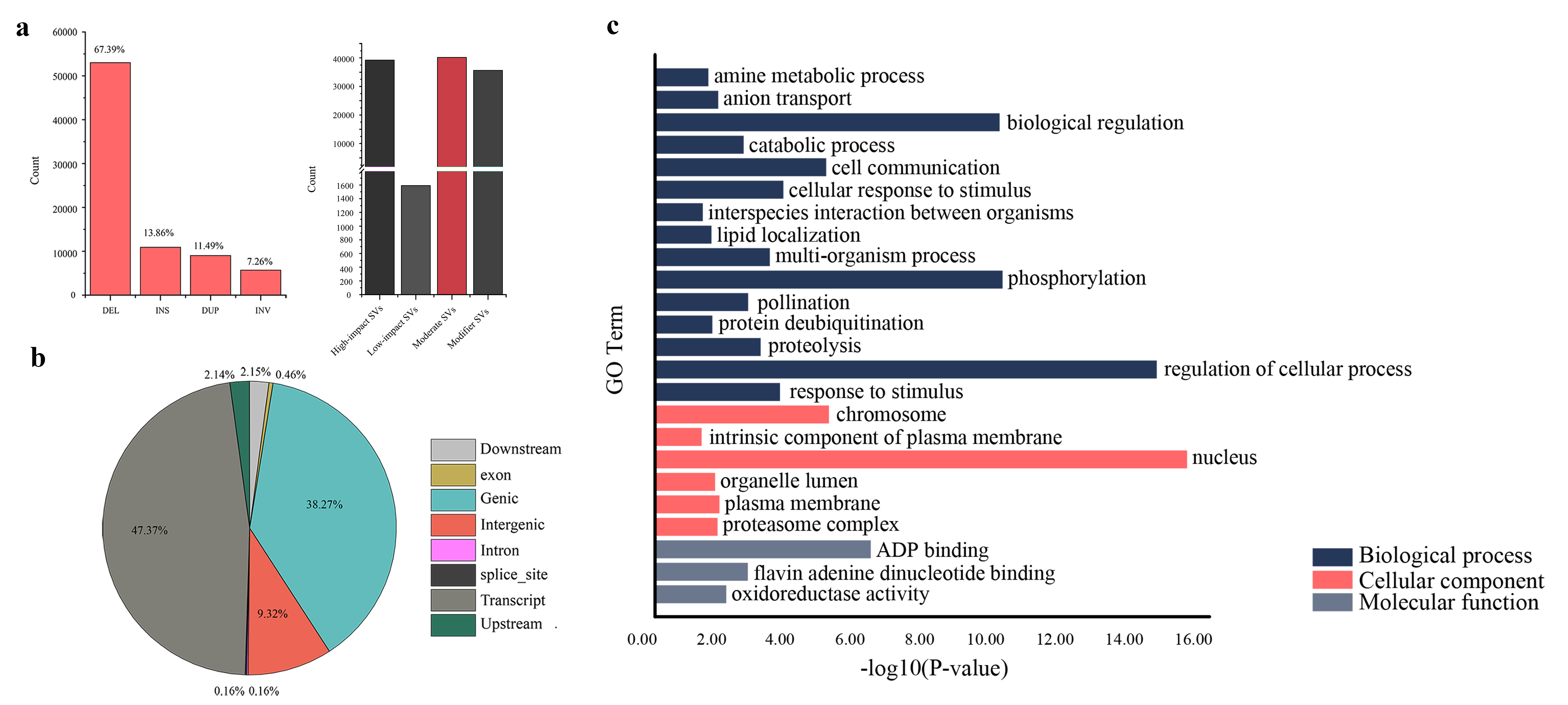
 **S9.** SVs and their functional enrichment. The SVs in the R108 genome were identified based on short read alignment using the A17 genome as a reference.

(a) Number of the R108-specific SVs, including deletions (DEL), insertions (INS), duplications (DUP), and inversions (INV), and number of the four categories of SV effects. (b) Annotation of all SVs categorized using SnpEff based on their position in the annotated R108 genome. ‘Downstream’ indicates the SVs located within 5 kb downstream from a gene; ‘Intergenic’ indicates the SVs in the intergenic region; ‘Splice_site’ indicates a splice variant that changes the 2 bp region at the 3' or 5' end of an intron; ‘Transcript’ indicates a feature ablation whereby the deleted region includes a transcript feature; ‘Upstream’ indicates the SVs located within 5 kb upstream of a gene. ‘Genic’ indicates genes whose function is predicted to be affected by the SVs. (c) GO enrichment of the high-impact SVs genes.

**Figure S10.** A Venn diagram showing the shared and specific PAVs detected by the short reads alignment and whole-genome alignment
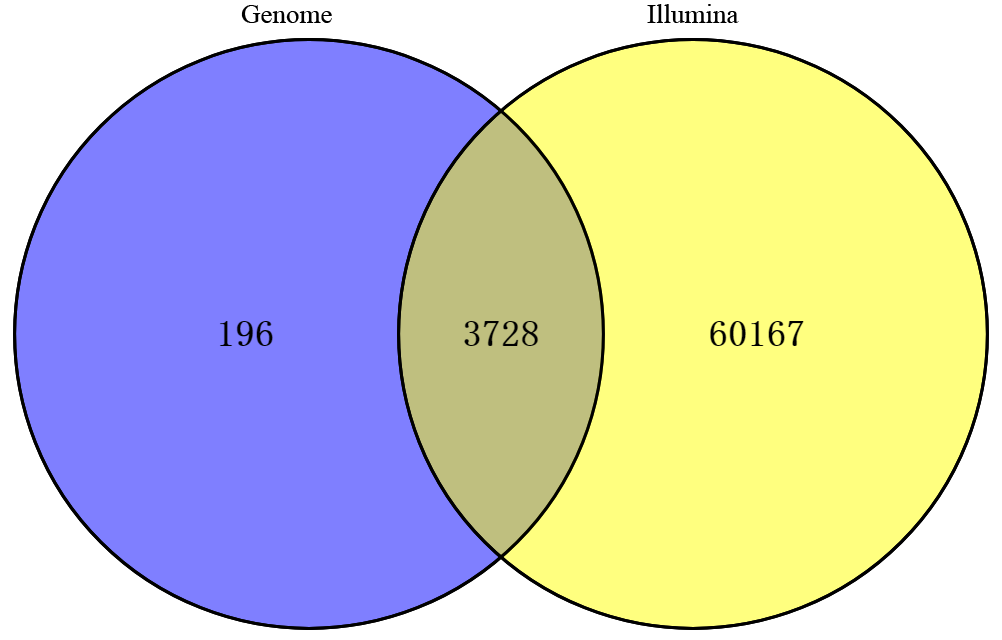
 between A17 and R108.


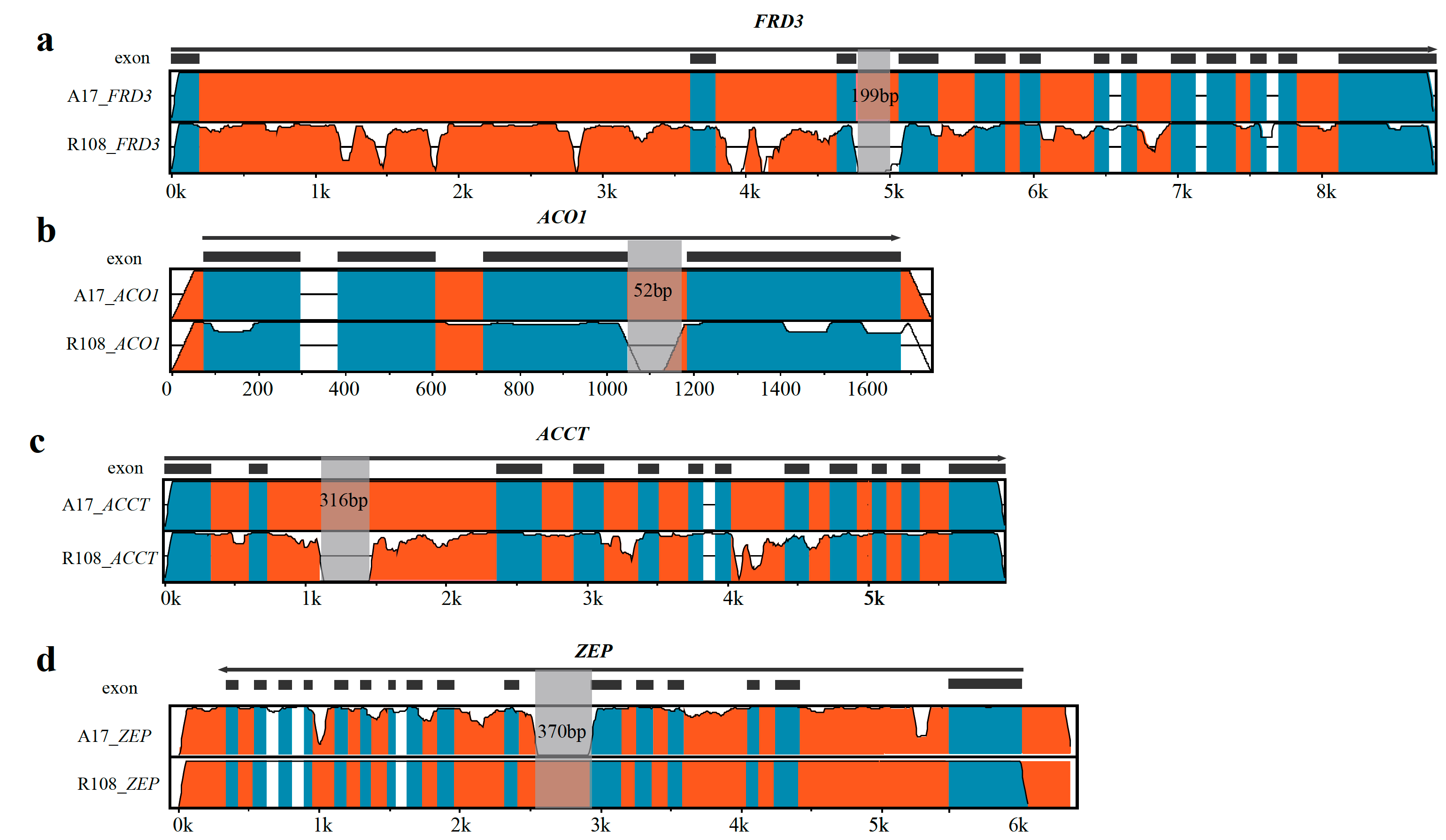


**Figure S11.** SVs in putative genes involved in the response to iron deficiency (a and b) aluminum toxicity (c), and drought stress (d) in A17 and R108. The gray areas indicate the SVs.

**Figure S12.**
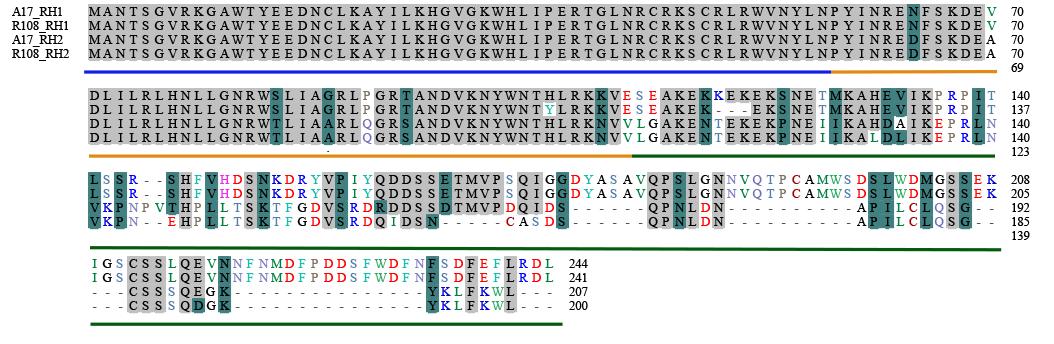
The protein sequences of the *RH1* and *RH2* genes in A17 and R108.

The blue and orange underline indicates the R2 repeat domain (R2 domain) and R3 repeat domain (R3 domain), respectively. The green underline indicates the C-terminal domain (CTD).

**Figure S13.** The distribution of NCR genes throughout
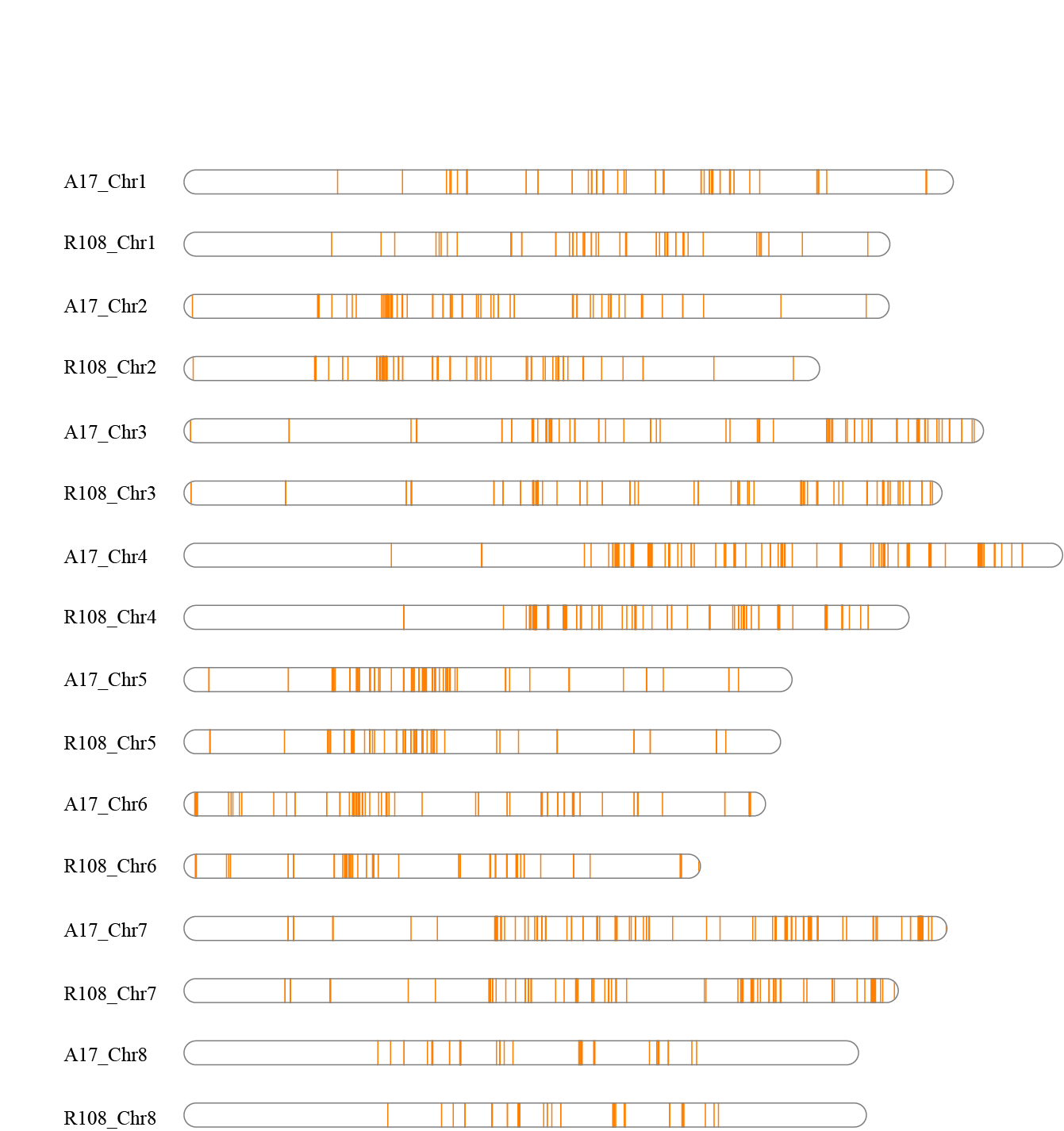
 the A17 and R108 genomes.
